# Surveillance Strategies for Sarcoma: Results of a Survey of Members of the Musculoskeletal Tumor Society

**DOI:** 10.1155/2016/8289509

**Published:** 2016-07-13

**Authors:** David D. Greenberg, Brooke Crawford

**Affiliations:** Saint Louis University Hospital, 7th Floor, Desloge Towers, 3635 Vista Avenue, St. Louis, MO 63110, USA

## Abstract

*Background*. Surveillance is crucial to oncology, yet there is scant evidence to guide strategies.* Purpose*. This survey identified sarcoma surveillance strategies for Musculoskeletal Tumor Society (MSTS) members and rationales behind them. Understanding current practice should facilitate studies to generate evidence-based surveillance protocols.* Methods*. Permission was granted by the Research and Executive Committee of the MSTS to survey members on surveillance strategies. First, the questionnaire requested demographic and clinical practice information. Second, the survey focused on clinicians' specific surveillance soft tissue and bone sarcoma protocols.* Results*. 20 percent of MSTS members completed the survey. The primary rationale for protocols was training continuation, followed by published guidelines, and finally personal interpretation of the literature. 95% of the respondents believe that additional studies regarding appropriate surveillance protocols are needed. 87% reported patient concerns regarding radiation exposure from surveillance imaging. For soft tissue and bone sarcoma local recurrence, responders identified surgical margin, histologic grade, and tumor size as the most important factors. For metastases, important risk factors identified included histologic grade, tumor size, and histologic type. Protocols demonstrated wide variation.* Conclusion*. This survey demonstrates that surveillance strategies utilized by MSTS members are not evidence-based, providing rationale for multi-institutional studies. It also confirms the public health issue of excessive radiation exposure.

## 1. Introduction

Surveillance to detect local or distant recurrence, identify adverse effects of treatment, and extend disease-free survival is an important component of oncology. Despite the recognized significance, there is a paucity of evidence to guide the intensity of surveillance strategies. The duration of surveillance needs to continue throughout the period of time in which the tumor is likely to recur. This potentially prolonged period creates substantial clinical and fiscal considerations. An ideal surveillance strategy should be easy to implement, accurate, and cost-effective [1].

Prior investigations support the notion that current follow-up strategies vary widely, are controversial and ill-defined, and lack evidence-based guidance [2–5]. Furthermore, the matters of cost and excessive radiation exposure are public health issues gaining national attention. Estimates that 1.5–2% of all cancers in the United States may be attributable to radiation from computed tomography (CT) studies have intensified the concern for unnecessary testing [6].

Surveys of members of the Society of Surgical Oncology (SSO) and United Kingdom (UK) clinicians who manage sarcomas have demonstrated significant variation in surveillance strategies and costs [6, 7]. However, orthopaedic oncologists comprise only 5% of SSO membership and practice patterns in the United States and UK are distinct.

The purpose of this study was to determine the current sarcoma surveillance strategies of members of the Musculoskeletal Tumor Society (MSTS) and the rationale behind them. Greater understanding of the current practice should facilitate future studies to generate the evidence-based surveillance protocols currently lacking.

## 2. Materials and Methods

Permission was granted by the Research and Executive Committee of the MSTS to survey members on their surveillance strategies. All members of the MSTS with a known email address were asked to participate in the survey through the MSTS email list. Qualtrics Survey Software (Qualtrics, LLC; Provo, Utah) was utilized to generate an online survey and a direct link to the survey was emailed to MSTS members.

The survey consisted of three parts. The first portion of the questionnaire requested physician demographic and clinical practice information. Additional questions focused on the rationale for the clinician's surveillance protocol and gauged perception of current surveillance guidelines. Questions regarding clinician and/or patient concerns for radiation exposure were also included in this section. The second part of the survey focused on soft tissue sarcomas (STS). Clinicians were asked to rank factors they considered to increase the risk of local recurrence or distant metastases. Charts were also provided to outline the clinician's specific surveillance protocol for STS. These charts were divided into soft tissue sarcomas believed to be at low or high risk for relapse. The final question in the second part asked whether early detection of metastases leads to improved survival in patients with STS. The third part of the survey was similar to the second but the focus was on bone sarcomas rather than STS. Clinicians were asked to complete this portion of the survey if their surveillance strategy differed for bone and soft tissue sarcomas.

After several email reminders with a survey link were distributed, the survey was closed and the data was analyzed using Qualtrics Survey Software.

## 3. Results

**Figure 1:**
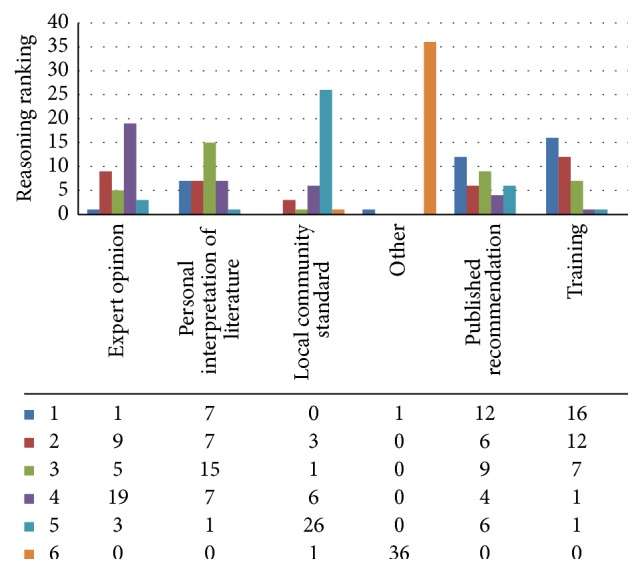
Reasoning behind surveillance strategies. Respondents numerically ranked reasoning options 1–6, with 1 being the most important reason supporting surveillance protocol. The majority (16 of 36 respondents) ranked continuation of the protocol used during training as the most important reason for current surveillance strategy. For those noting published recommendations, all referenced NCCN guidelines as their resource, with one respondent also referencing COG.

20 percent (38 of 193) of MSTS members completed the survey. The demographics profile for all respondents is presented in Table 1. The reasoning responders provided for surveillance is depicted in Figure 1. The most common rationale for the specific protocol utilized was continuation of the protocol used during training. The second most important was recommendations from published guidelines (National Comprehensive Cancer Network [NCCN]) followed by personal interpretation of available literature. When asked whether there is a need for additional studies regarding appropriate surveillance protocols, 95% of respondents answered in the affirmative and stated they would be willing to enroll their patients into such trials. Patients may also be interested in surveillance studies as 33 respondents (87%) reported that their patients expressed concern regarding radiation exposure from surveillance imaging. Greater than half of responding physicians (56%) have changed their surveillance protocol in response to these concerns.

**Figure 2:**
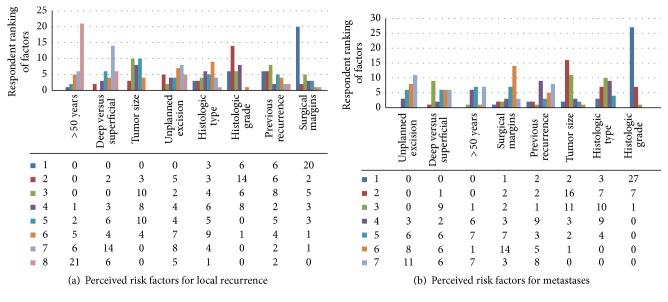
(a) Respondents were asked to rank 8 factors according to contribution to risk of local recurrence. The factor ranked 1 was perceived to most significantly increase risk of local recurrence. Of the 8 factors listed, respondents felt surgical margin was the factor that contributed most to local recurrence (20 ranked it as #1), followed by histologic grade. Age was the factor ranked lowest by survey participants (21 ranked it #8). (b) Respondents were asked to rank 8 factors according to contribution to risk of metastases. The factor ranked 1 was perceived to most significantly increase risk of metastasis. The most important risk factor for metastatic disease was felt to be histologic grade (27 respondents ranked it #1), followed by tumor size and histologic type. Unplanned excision before referral was perceived to contribute the least to subsequent metastatic disease.

In the soft tissue sarcoma section, responders were surveyed regarding their perceived risk factors for relapse. Eight factors were provided for consideration of risk of local recurrence (Figure 2(a)). From the 35 respondents who answered the question, the three factors most commonly selected were surgical margin, histologic grade, and tumor size. Of note, age over 50 years was the factor perceived to be the least significant. In regard to risk of metastatic disease, Figure 2(b) depicts the provided factors. Histologic grade was overwhelmingly chosen as the factor that most significantly increases the risk of metastases (mean ranking of 1.26 out of the eight factors), followed by tumor size and histologic type. Here again, age over 50 years was selected as the least significant. Surgical margin had a mean ranking of 5.34 regarding significance for risk of metastases.

MRI was the modality most frequently utilized for detection of local recurrence of a soft tissue sarcoma. For a STS considered at* low risk* of relapse, an average of 1.68 MRI tests (mode: 2; range: 0–4) were ordered during the first year of surveillance. For soft tissue sarcomas considered* high risk* for recurrence, an average of 2.08 MRI tests (mode: 2; range: 0–4) were ordered during the first year of surveillance. The final question in the soft tissue sarcoma section asked whether early detection of metastases leads to improved survival in patients with STS. Responses were yes (41%), no (27%), or do not know (32%).

Bone sarcoma surveillance was generally similar to the soft tissue sarcoma protocols utilized by the responders of the survey. 34 respondents (92%) reported similar systemic monitoring (one respondent did not complete this section of the survey). X-ray of the primary site was the modality frequently utilized for local imaging for bone sarcomas as opposed to MRI for STS. More respondents felt that early detection of metastases leads to improved survival in patients with bone sarcoma as compared to soft tissue sarcomas (67% yes, 19% no, and 14% do not know).

**Figure 3:**
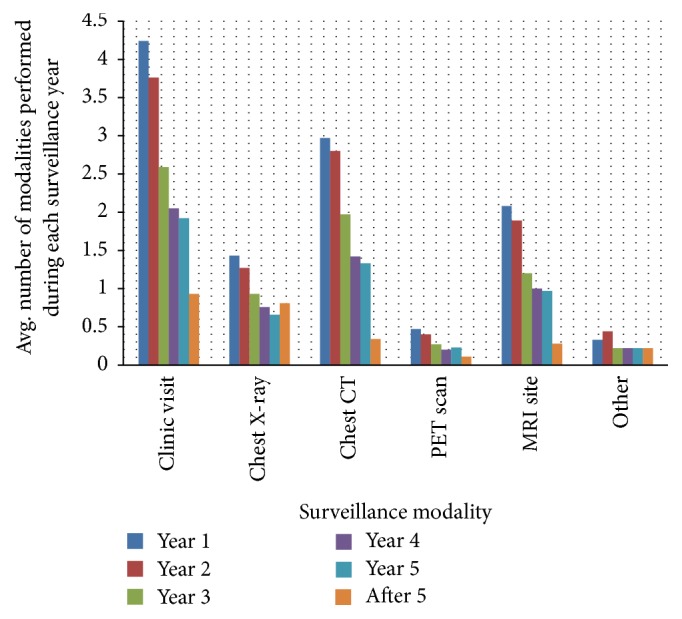
Surveillance protocols. Average surveillance strategies based on 38 respondents depicted as the average number of all modalities performed during each year of surveillance. Chest CT was more common for surveillance as compared to chest X-ray by an approximate 2 : 1 ratio in years 1–3, with reversal in strategy after year 5 with X-ray becoming more common by an 8 : 3 ratio.

The actual surveillance protocols utilized by the respondents demonstrated wide variation during the course of follow-up (Figure 3). In the first year of surveillance, the range of clinic visits for soft tissue sarcoma was 2–12, with 4 visits being the most common pattern. Regarding chest imaging during the first two years of surveillance for STS considered at high risk of local or systemic relapse, the number of chest X-rays ranged from 0 to 13 and the number of chest CT scans ranged from 1 to 8. The most common pattern reported (46% of respondents) was 4 chest CT scans in each of the first two years of surveillance then tapering thereafter. Survey respondents also varied considerably in how long they performed surveillance on STS patients, with 19% following up for a lifetime, 32% following up for 1–5 years, and 43% following up for 5–10 years. With bone sarcomas, first-year surveillance clinic visits ranged from 3 to 6, chest X-rays ranged from 0 to 3, chest CT scans ranged from 1 to 4, and X-ray of the primary site ranged from 3 to 6. 65% of respondents reported following up bone sarcoma patients for life with the remaining 35% selecting 5–10 years.

## 4. Discussion 

Surveillance is an important component of patient care after completion of definitive treatment. The factors to consider when designing a surveillance protocol should include the following: (1) the interval between examinations and duration of testing should be consistent with time of maximal risk of recurrence; (2) tests should focus on the most likely recurrence locations and have high positive and negative predictive values; (3) there should be a possibility of cure, prolongation of life, or palliation; and (4) risk of secondary malignancy should be considered when ordering tests [8]. It is unclear whether all of these goals are achievable for sarcoma, as illustrated by only 41% of respondents to this study believing that early detection of metastases leads to improved survival in patients with STS. However, almost all patients treated for sarcoma undergo some level of surveillance and 95% of those surveyed believe that efforts to achieve these goals need to be evidence-based. The results of this survey demonstrate that current surveillance strategies utilized by members of the MSTS are not guided by high-quality evidence and demonstrate wide variation, with most continuing the protocol taught during fellowship training. The findings lend credence to prior articles that express concerns regarding the quality of evidence underlying surveillance rationale [1, 3, 6, 7].

This survey study is not without limitations. Most concerning is the low percentage of respondents with only 20% of MSTS members completing the survey. This fairly low rate of response generates the potential for significant nonresponse bias. Counteracting this weakness is the fact that the responders were entirely made up of a population previously underrepresented in surveillance surveys. Beitler et al. published results of follow-up strategies of members of the Society of Surgical Oncology [2]; however, orthopaedic oncologists comprised only 5% of the responders. Only 30 of 121 survey respondents were orthopaedic surgeons in a similar follow-up survey in the United Kingdom [7]. One hundred percent of responders in the current survey were orthopaedic surgeons. Furthermore, members of the MSTS represent a significant portion of sarcoma care providers in the United States and surveillance data on this membership has not been previously reported. Another limitation is the generalizability of this data. Current surveillance strategies of MSTS members may not be reflective of sarcoma care nationally or internationally. However, the fact that our survey results are similar to previously reported SSO and UK surveys does support the translatability of the findings.

A key finding in both the current survey and previous sarcoma surveillance surveys is the wide variation among respondents' protocols. This was illustrated most simply in the UK survey by the cost analysis, with low risk trunk/extremity tumor surveillance costs ranging from 372 to 7,852 pounds and high risk tumor surveillance costs ranging from 1,091 to 7,961 pounds [7]. Our survey showed wide ranges in the frequencies of modalities utilized: for example, first-year surveillance clinic visits ranged from 2 to 12, chest radiographs ranged from 0 to 9, and chest CTs ranged from 0 to 4. While these ranges may fall within the current NCCN guidelines for sarcoma follow-up, the cost and radiation exposure differences are substantial.

NCCN guidelines are most commonly utilized by responders of this survey. The NCCN sarcoma follow-up guidelines call for chest radiograph or chest CT every 3–6 months for 2-3 years and then every 6 months for the next 2 years and then annually [1]. This guideline allows for tremendous variation in frequency and modality of chest imaging for sarcoma surveillance, a finding reflected in the results of this survey. As stated above, this variation has important implications regarding not only cost but also radiation exposure for the patient. This survey confirmed that the public health issue of excessive radiation exposure is something patients are aware of [2, 9], with 87% of respondents reporting having patients express concern. The fact that over half of survey respondents have changed their protocol in response is also alarming as reaction to patient anxiety is not better justification for surveillance strategies than the empiric rationale currently utilized.

Puri et al. in a randomized noninferiority trial demonstrated that chest radiography did not lead to worsened survival and was not inferior to chest CT scan in terms of detecting pulmonary metastasis during sarcoma surveillance [5]. Our survey revealed significant utilization of chest CT for pulmonary surveillance, especially during the first two years after treatment. The fiscal and radiation reduction gained by converting from chest CT to chest radiography has been highlighted previously [1, 4, 7]. It is not the intention nor is it within the scope of a survey study to outline an appropriate surveillance strategy for sarcomas. However, this survey does establish that there is interest among respondents in pursuing evidence-based guidelines and willingness to enroll patients in such a trial.

The results of this study illustrate that in general the issues in sarcoma surveillance are not unique to any one subspecialty but rather universal to all clinicians following up sarcoma patients. One key difference was identified specific to orthopaedic oncologists in their surveillance protocols as compared to multispecialty surveys done in the United Kingdom and among SSO members. Chest CT is the surveillance modality most commonly used by the MSTS respondents for the first five years posttreatment. In the UK survey, >85% of those surveyed used chest X-rays as primary imaging for low and high risk trunk or extremity tumors, whereas only 16% used chest CT scans for low grade tumors and 29% used them for high grade tumors [7]. In an SSO survey, Johnson et al. showed that chest X-ray was used more than chest CT across metropolitan areas, in managed care organizations, and by US Census Regions [3]. Neither of these surveys identified differences in practices between specialties treating sarcomas.

Perceived risk factors between MSTS respondents and those surveyed in the United Kingdom were similar, with both groups ranking surgical margin and histologic grade as the most important risk factors for recurrence and histologic grade and tumor size for metastasis. This question was not addressed in the SSO survey.

In conclusion, this study confirms that the wide variation in surveillance practice demonstrated in prior studies also exists among members of the MSTS. High-quality evidence for guidance is currently absent but the need for it is recognized and warranted. Combined, these factors provide sound rationale for a multi-institutional study on appropriate sarcoma surveillance.

## Figures and Tables

**Table 1:** The demographic data obtained from the MSTS member respondents.

*Specialty (%)*	*Orthopaedic surgery*	*General surgery*	*Medical oncology*	*Radiation oncology*	
	38 (100)	0	0	0	

*Country of practice (%)*	*USA*	*Canada*	*Other*		
	34 (89)	3 (8)	1 (3)		

*Age (%)*	*<30*	*30–40*	*41–50*	*51–60*	*>60*
	0	22 (58)	7 (18)	7 (18)	2 (5)

*Gender (%)*	*Male*	*Female*			
	79	21			

*Length of career (%)*	*0*–*5*	*6*–*10*	*11*–*15*	*16*–*20*	*>20*
	16 (42)	9 (24)	4 (11)	1 (3)	8 (21)

*# ST sarcomas/yr (%)*	*<10*	*10*–*30*	*>30*		
	1 (3)	22 (58)	15 (39)		

*# bone sarcomas/yr (%)*	*0*–*5*	*5*–*10*	*10*–*20*	*>20*	
	3 (8)	18 (47)	12 (32)	5 (13)	

*Multidisciplinary sarcoma team (%)*	*Yes*	*No*			
	95	5			
